# Thirty‐day readmission after spontaneous intracerebral hemorrhage

**DOI:** 10.1002/brb3.935

**Published:** 2018-02-09

**Authors:** Anna Therese Bjerkreim, Andrej Netland Khanevski, Solveig Bergliot Glad, Lars Thomassen, Halvor Naess, Nicola Logallo

**Affiliations:** ^1^ Department of Clinical Medicine University of Bergen Bergen Norway; ^2^ Department of Neurology Haukeland University Hospital Bergen Norway; ^3^ Norwegian Health Association Oslo Norway; ^4^ Centre for age‐related medicine Stavanger University Hospital Stavanger Norway; ^5^ Department of Neurosurgery Haukeland University Hospital Bergen Norway

**Keywords:** hospital readmission, intracerebral hemorrhage, outcomes, stroke

## Abstract

**Background:**

Intracerebral hemorrhage (ICH) is the most severe form of stroke, but data on readmission after ICH are sparse. We aimed to determine frequency, causes, and predictors of 30‐day readmission after ICH.

**Materials and Methods:**

This retrospective cohort study includes all spontaneous ICH survivors admitted to the stroke unit at Haukeland University Hospital in Bergen in Norway from July 2007 to December 2013. Patients were followed by review of electronic medical charts, and the first unplanned readmission within 30 days after discharge was used as final outcome. Cox regression analysis was performed to identify predictors of 30‐day readmission.

**Results:**

We identified 226 patients with spontaneous ICH, 70 (31.0%) of whom died before discharge or were discharged to palliative care. Of the remaining 156 ICH survivors, 28 (18.0%) were readmitted within 30 days. Median time to readmission was 12 days (IQR 4.5 – 18.5). Most patients were readmitted due to infections (*N* = 13). None of the patients were readmitted with recurrent stroke. Pneumonia and enteral feeding during the index hospitalization were associated with readmission for infections (both *p *<* *.01). Age was the only independent predictor of readmission (HR 1.06, 95% CI 1.02 – 1.11, *p *=* *.006).

**Conclusions:**

Almost one in five of our spontaneous ICH survivors was readmitted within 30 days, and most readmissions were caused by infections.

## 1 INTRODUCTION

Intracerebral hemorrhage (ICH) is the most severe form of stroke with the highest rates of dependence and death (Dennis, 2003; Weimar et al., 2003). Up to 85% of all stroke patients experience complications (Bovim, Askim, Lydersen, Fjaertoft, & Indredavik, 2016; Indredavik, Rohweder, Naalsund, & Lydersen, 2008), including pneumonia, urinary tract infections, fractures, and progressing stroke (Bovim et al., 2016; Indredavik et al., 2008; Otite et al., 2017). Despite this, in‐hospital mortality is decreasing (Bejot et al., 2017; Otite et al., 2017), resulting in more patients available for readmission. Complications during stroke admission are associated with 30‐day readmission (Shah et al., 2015). Most studies on 30‐day readmission after stroke have focused on ischemic stroke, and there is sparse research on readmission after ICH. Frequencies of 30‐day readmission after ICH range from 11% to 16% (Lichtman, Jones, Leifheit‐Limson, Wang, & Goldstein, 2011; Liotta et al., 2013; Lord et al., 2016), and opposite to ischemic stroke where many readmissions are caused by new cardiovascular events, most readmissions after ICH are caused by infections (Lord et al., 2016).

We performed a retrospective cohort study with the aim to determine frequencies, causes, and predictors of 30‐day readmission after ICH.

## 2 MATERIALS AND METHODS

All stroke patients admitted to the stroke unit at Haukeland University Hospital between 1 July 2007 and 31 December 2013 were prospectively registered in the Bergen NORSTROKE Registry. The stroke unit serves a well‐defined geographical area of approximately 275,000 people. Patients living outside this area were excluded from the study. Stroke was defined as “rapidly developed clinical signs of focal (at times global) disturbances of cerebral function, lasting more than 24 hr or leading to death with no apparent cause other than that of vascular origin” (Hatano, 1976). Stroke subtype was assessed by review of clinical and radiological findings by an experienced neurologist (HN). Spontaneous ICH was defined as intracerebral hematoma on CT or MRI. Traumatic ICH, extracerebral intracranial hemorrhages, hemorrhagic infarctions, and ICH related to neoplasia or thrombolytic treatment were excluded. Hematoma volume was calculated with the A × B × C/2 formula (Kothari et al., 1996). Stroke severity on admission and on day 7 was assessed by the National Institutes of Health Stroke Scale (NIHSS) score. Short‐term functional outcome was determined on day 7, or at discharge, if earlier, by modified Rankin Scale (mRS) score and Barthel Index (BI) score. Clinical characteristics, treatment, medical history, comorbidity, complications, and discharge destination were registered during hospitalization. Secondary preventive treatment from the day of discharge was based on the Norwegian guidelines for stroke treatment (Helsedirektoratet, 2010).

Two clinicians (ATB and AK) collected information on readmission and death by reviewing electronic medical charts from all hospitals in our region (Western Norway Regional Health Authorities) with approximately 1.1 million inhabitants and nine operating hospitals. The study endpoint was the first readmission within 30 days after discharge. Readmission was defined as an unplanned (emergency) admission to any hospital department. Patients who died during index hospitalization or were discharged to palliative care were excluded from the analyses (Lichtman et al., 2010). Association between complications with infection or enteral feeding (nasogastric tube or percutaneous endoscopic gastrostomy) during index hospitalization and infection‐related readmission was assessed by comparing patients readmitted with infection to all other patients not readmitted with infection.

We obtained written informed consent from all the patients or their legally authorized representatives. The study was approved by the Western Regional Ethics Committee.

### 2.1 Statistics

Baseline characteristics were investigated by chi‐squared test for categorical variables and Student's *t* test or Mann–Whitney *U* test for the continuous variables when appropriate. Cox regression analysis was used to investigate predictors of 30‐day readmission. Age, sex, mRS score, and length of stay were forced into the regression analyses to adjust for potentially confounding factors. Statistical analyses were performed using Stata 14.0 (Stata Corporation, College Station, TX, USA).

## 3 RESULTS

Among 2216 stroke patients, 226 patients presented with spontaneous ICH (10.2%), 67 (29.7%) of whom died in the stroke unit, and three (1.3%) were discharged to palliative care. The final study cohort consisted of 156 spontaneous ICH survivors, 28 (18.0%) of whom were readmitted within 30 days after discharge. Two patients were readmitted twice within 30 days. Median days from discharge to readmission were 12 (interquartile range 4.5–18.5). The baseline characteristics of the cohort are described in Table 1. In univariate analyses, readmitted patients were older, had a higher NIHSS score at admission, and a higher occurrence of pneumonia during the index admission than patients who were not readmitted within 30 days. Although not statistically significant, readmitted patients had poorer functional outcome (higher mRS score and lower BI score), and more frequently experienced complications during index admission, and were more often institutionalized after discharge from the stroke unit than patients who were not readmitted within 30 days. Age was the only independent predictor of 30‐day readmission (HR = 1.06, 95% CI = 1.01 – 1.11, *p *=* *.010).

**Table 1:** Baseline Characteristics of Study Population Stratified by 30‐day Readmission

	Readmitted *N* = 28	Not readmitted *N* = 128	*p*
Demographics			
Age (years), mean ± *SD*	78.1 ± 10.6	71.1 ± 13.2	<.01
Male sex	15 (53.6)	72 (56.3)	.80
Stroke severity and functional outcome			
NIHSS score on admission, median (IQR)	8 (3, 16)	6 (2, 11)	.05
NIHSS score at discharge, median (IQR)	6 (2, 11)	5 (2, 8.5)	.19
mRS score at discharge, median (IQR)	4 (3, 4)	3 (2, 4)	.25
BI score at discharge, median (IQR)	35 (17.5, 82.5)	65 (25, 100)	.10
ICH volume (ml), median (IQR)	13.5 (4.3, 28.2)	6.9 (2.8, 25.7)	.29
ICH location			.14
Lobar	6 (33.3)	50 (52.1)	
Basal ganglia	12 (66.7)	46 (47.9)	
Comorbidities			
Cerebrovascular disease	6 (21.4)	22 (17.2)	.60
Diabetes	1 (3.6)	13 (10.2)	.27
Hypertension	11 (39.3)	71 (55.5)	.12
Atrial fibrillation	9 (32.1)	23 (18.0)	.09
Prior/current smoking	13 (46.4)	71 (55.5)	.39
Coronary artery disease	5 (17.9)	26 (20.3)	.77
Medication prior to index admission			
Reported anticoagulant use	7 (25.0)	17 (13.3)	.12
Reported ASA‐ERDP use	7 (25.0)	38 (29.7)	.62
Complications during ICH hospitalization			
Urinary tract infection	8 (28.6)	33 (25.8)	.76
Urinary incontinence	6 (21.4)	31 (24.2)	.75
Urinary retention	14 (50.0)	56 (43.8)	.55
Pneumonia	11 (39.3)	24 (18.8)	.02
Enteral feeding^a^	9 (32.1)	25 (19.5)	.14
Seizures	3 (10.7)	12 (9.4)	.83
Stroke in progression	5 (17.9)	24 (18.8)	.91
Any complication	21 (75.0)	81 (63.3)	.24
Length of stay, median (IQR)	13.5 (7.5, 20)	10 (5.5, 17.5)	.29
Discharge destination			.19
Home	5 (17.9)	35 (27.3)	
Home nursing	2 (7.1)	15 (11.7)	
Rehabilitation	3 (10.7)	28 (21.9)	
Nursing home	17 (60.7)	46 (35.9)	
Other departments	1 (3.6)	4 (3.1)	

*SD*, standard deviation; mRS, modified Rankin Scale; IQR, interquartile range; NIHSS, National Institutes of Health Stroke Scale; BI, Barthel Index; ICH, intracerebral hemorrhage; ASA‐ERDP, aspirin plus extended‐release dipyridamole. Data are expressed as *N* (%) unless specified.

a Nasogastric tube or percutaneous endoscopic gastrostomy.

Main causes of 30‐day readmission are presented in Table 2. Among all the ICH survivors, 13 (8.3%) were readmitted with infections. The most common infection was pneumonia (*n* = 6), of whom five had pneumonia during index hospitalization. Patients who developed infections during index hospitalization were more likely to be readmitted with an infection compared to patients without infections during index admission (15.4% vs. 3.3%, *p *<* *.01). Patients who received enteral feeding (nasogastric tube or percutaneous endoscopic gastrostomy) during index hospitalization were also more prone to be readmitted with infection (20.6% vs. 4.9%, *p *<* *.01). Two patients were readmitted with epileptic seizures. No patients, however, were readmitted with recurrent stroke.

**Table 2:** Causes of 30‐day unplanned readmission after ICH

Readmission diagnosis	*N*
Cardiovascular disease	1
Recurrent stroke	0
Venous thromboembolism	1
Aggravation of neurological deficits	2
Seizure	2
Hip Fracture	1
Infection	13
Pneumonia	6
Urinary tract infection	1
Sepsis	5
Gastrointestinal infection	1
Other causes	8

ICH, intracerebral hemorrhage.

## 4 DISCUSSION

We found that 18% of all ICH survivors were readmitted within 30 days after discharge. This is a higher rate than what has been reported by a similar single‐center study (11%) (Liotta et al., 2013), but is comparable with two large cohort studies from the United States (14.5% – 16.0%) (Lichtman et al., 2011; Lord et al., 2016).

As previously reported from Italy and the United States (Liotta et al., 2013; Lord et al., 2016), the most frequent cause of readmission was infections, which accounted for almost half of all the readmissions in our study. We have previously reported that 26% of all ICH readmissions within 90 days are caused by infections (Bjerkreim, Thomassen, Waje‐Andreassen, Selvik, & Naess, 2016). This indicates that most poststroke infections leading to readmission happen within the first 30 days after discharge.

Poststroke immunosuppression likely contributes to the high rate of infection‐related readmissions (Chamorro, Urra, & Planas, 2007; Sykora et al., 2011). In addition, large lesion volume and stroke severity are reported to be associated with infections (Urra et al., 2017). This is probably due to immobilization, invasive procedures such as placement of urinary catheters and feeding tubes, and neurological deficits such as dysphagia and reduced consciousness, which may cause aspiration. We found that infections and enteral feeding during index admission were both associated with infection‐related readmissions. Enteral feeding is associated with the development of poststroke pneumonia (Brogan, Langdon, Brookes, Budgeon, & Blacker, 2014), and poststroke pneumonia is in turn associated with poor functional outcome and higher mortality (Finlayson et al., 2011; Katzan, Cebul, Husak, Dawson, & Baker, 2003). Although most readmissions after stroke may be unavoidable, close postdischarge surveillance, especially in patients receiving enteral feeding or who develops infections during index hospitalization, may prevent some readmissions related to infection.

A limitation of our study is the low number of patients included. ICH is a relatively rare condition, thus resulting in a small cohort even though patients were collected over several years from a large Norwegian stroke center. A larger cohort is needed to have statistical power for risk factor identification. Large studies are, however, often based on administrative databases which do not have the same data verification of clinical characteristics compared to data collected by medical chart reviews as in our study. The single‐hospital design may cause selection bias, and caution is needed when generalizing our findings.

In conclusion, unplanned readmission of ICH survivors within 30 days is mostly due to infections. Our study indicates that preventing, detecting, and treating infections aggressively before discharge might be important to prevent readmission after ICH.

## CONFLICT OF INTERESTS

The author(s) declare no potential conflict of interests with respect to the research, authorship, and/or publication of this article.
